# Co-existence of well-differentiated fetal adenocarcinoma of the lung with tuberculosis in a young female: A rare case report

**DOI:** 10.1097/MD.0000000000018282

**Published:** 2019-12-10

**Authors:** Fengzhu Guo, Jiantao Wang, Haoyue Hu, Xiaoxiao Xie, Kuncheng Liu, Feng Luo

**Affiliations:** a Lung Cancer Center, Cancer Center, State Key Laboratory of Biotherapy, West China Hospital of Sichuan University; b Department of Neurology, West China Hospital, Sichuan University, Chengdu, Sichuan, China.

**Keywords:** differential diagnosis, tuberculosis, well-differentiated fetal adenocarcinoma

## Abstract

**Rationale:**

Fetal adenocarcinoma of the lung (FLAC) with fetal lung-like morphology is a rare entity of pulmonary adenocarcinoma. Well-differentiated fetal adenocarcinoma (WDFA) belongs to its the low-grade form, which possesses a relatively favorable prognosis. Tuberculosis (TB) is an aggressive infectious disease that has been ranked as one of the top 10 causes of death worldwide. There may be a connection between the 2 and attention should be paid to the differential diagnosis.

**Patient concerns:**

A 28-year-old non-smoking female was admitted with signs of hemoptysis, and she had been coughing up phlegm for 5 years. The patient was previously diagnosed with TB in another hospital, and underwent an anti-TB regimen.

**Diagnosis:**

The co-existence of WDFA and TB was confirmed via histopathological evaluation of postoperative samples.

**Interventions:**

The patient was subjected to a right lower lobectomy together with a wedge resection of the right upper lobe using video-assisted thoracoscopic surgery, with systemic lymphadenectomy.

**Outcomes:**

The patient tolerated the surgical procedure well and underwent an uneventful postoperative course.

**Lessons:**

To our knowledge, no previous reports exist of cases with WDFA accompanied by TB. The present case indicated that a prior diagnosis of TB might predispose to lung cancer regardless of smoking history. It is also essential to distinguish WDFA from TB because of the similarity in clinical features and sites of pathological changes. Patients with WDFA usually have a better prognosis and surgery is the preferred treatment.

## 1 Introduction

Fetal adenocarcinoma of the lung (FLAC), an extremely rare malignant lung tumor, is currently classified by World Health Organization (WHO) as a variant of invasive lung adenocarcinoma. By microscopic examination, FLAC is characterized by glycogen-rich neoplastic glands and tubules that resemble the airway epithelium in the pseudoglandular phase (8–16 weeks of gestation) of the fetal lung. FLAC has been further categorized into high-grade and low-grade forms.^[1]^ The low-grade form, also known as well-differentiated fetal adenocarcinoma (WDFA), is a distinctive malignancy constituting 0.1% or fewer across all primary pulmonary neoplasms.^[2,3]^

Furthermore, tuberculosis (TB) is a critical public health threat that affected approximately 10.0 million individuals in 2017. Worldwide, around 23% of the population is estimated to experience a latent TB bacilli infection, which may progress to active TB disease later in life.^[4]^ According to the WHO global TB report, China is listed as one of the most prevalent countries with a heavy burden of TB.^[4]^ In areas of high lung cancer and TB prevalence, the co-existence of both could be occasional, however, increasing evidence substantiates a possible causality between the WDFA and TB.^[5]^ In this study, a rare case of a young non-smoking female with both WDFA and TB is described.

## 2 Case presentation

**Figure 1:**
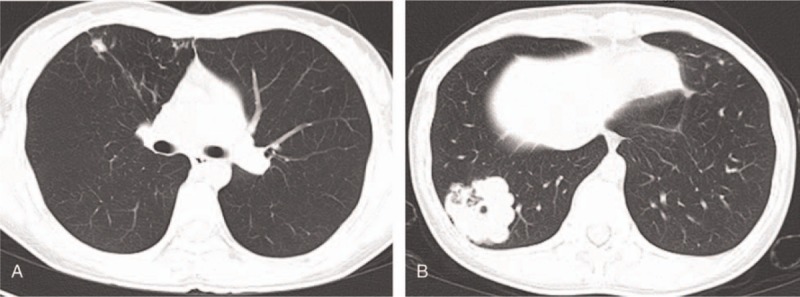
Computed tomography of the chest revealing (A) irregular nodular lesions in the right upper lobe and (B) a well-demarcated, solitary lung mass located in the right lower lobe peripherally.

A 28-year-old non-smoking female presented to our hospital with signs of hemoptysis and coughing up phlegm. Five years prior, the above-mentioned symptoms occurred, and according to sputum examination were followed by TB in the other hospital. The patient received anti-tubercular therapy for the duration of a year, and chest computed tomography (CT) showed nodules of 0.5 to 1.0 cm in the right lung, favoring pulmonary tuberculomas (Fig. 1A). For the following 6 months, she underwent continuous anti-tuberculosis treatment. Subsequently, the chest CT was repeated annually, and no significant changes in the nodules of the right lung were observed. One year before the current admission, she developed a cough, sputum, hot flashes, and night sweats, without hemoptysis or chest pain. CT exhibited patchy consolidations, and a lobular, well-circumscribed mass lesion with an irregular thick-walled cavity located in the right lower lobe lung (Fig. 1B), which was confirmed adenocarcinoma by a CT-guided biopsy. Moreover, scattered nodules and fibrous stripes were observed in the bilateral lungs. As for TB related tests, the sputum smear was negative for acid-fast staining. In addition, the results of the interferon-gamma release assay of TB infection (TB-IGRA (T-N)) was positive (98.76 pg/ml). Combined with laboratory and imaging findings, the patient had a TB infection but did not active TB at that time.

**Figure 2:**
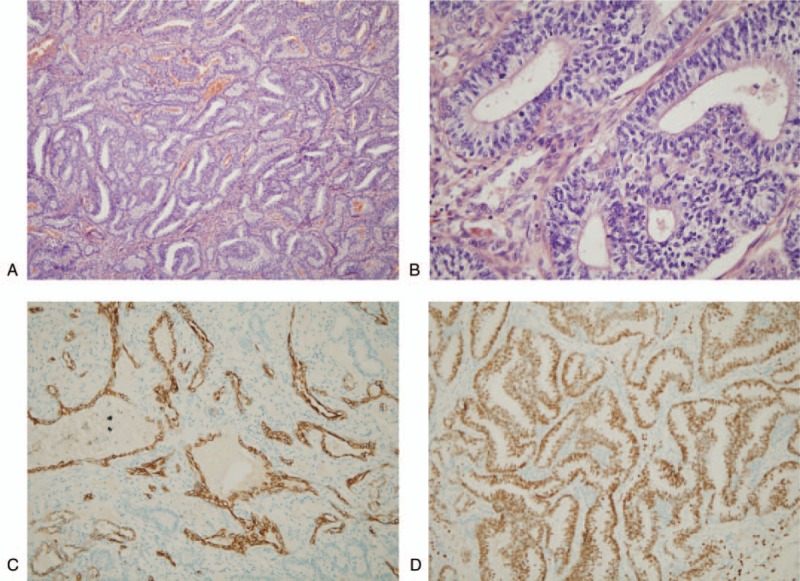
Pathological analysis demonstrating (a, 100× and b, 400×) glycogen-rich adenocarcinoma cells with mild nuclear atypia, prominent morule formation as well as characteristic subnuclear and/or supranuclear vacuoles. Immunohistochemical staining for (c, 200×) cytokeratin 7, and (d, 200×) thyroid transcription factor-1.

A right lower lobectomy combined with a wedge resection of the right upper lobe was performed using video-assisted thoracoscopic surgery, with harvesting of systemic lymph nodes. Surgical findings demonstrated a hard tumor, approximately 6.5 × 6 × 4 cm in size, associated with a fish-like cut surface. In addition, 2 and 3 nodules were observed in the right lower and right upper lobes, respectively. Our patient had an uneventful postoperative recovery without complications. The final histopathological diagnosis of the specimen confirmed WDFA (Fig. 2A and B). Immunohistochemical analysis of the neoplastic glands was positive for β-catenin, cytokeratin 7, thyroid transcription factor-1, and napsin A (focal) (Fig. 2C and D). Staining for estrogen receptors, myogenin, desmin, anaplastic lymphoma kinase-V, and S-100 protein was negative. According to postoperative pathological examination, the tumor stage was pT3N0M0 IIB. The nodules in the right upper lobe showed granulomatous inflammation with necrosis and calcification and a nodule was present containing acid-resistant positive bacilli. Then, multiple tuberculomas in the upper lobe of the right lung were added in the subsequent modified diagnosis. The patient was regularly evaluated in the Cancer Center and Department of Respiration, and did not receive postoperative adjuvant chemotherapy due to TB.

## 3 Discussion

FLAC represents a rare subtype of lung neoplasia that harbors characteristics concerning age of onset, risk factors in epidemiology.^[6]^ High-grade FLAC predominantly appears in elderly men while low-grade FLAC tends to occur in young women in the third to fourth decade of life.^[7]^ WDFA is a smoking-associated type of cancer.^[8]^ Patients with WDFA commonly present with non-specific clinical manifestations, including cough, sputum, hemoptysis, chest pain, and chest tightness among several unexpected findings.^[9]^ However, in this case, there was no history of smoking, and the remainder, such as the episode age and clinical manifestations were compatible with the data presented in previous studies. The patient was a young female, and with 28 years of age, she was close to the high-risk age range of WDFA. Cough and sputum were the main symptoms associated with WDFA, and were both common manifestations of respiratory diseases.

Regarding tumorigenesis, a prior diagnosis of TB may increase the risk of lung cancer regardless of smoking.^[10]^ The predisposition of TB to lung cancer has long been the focus of intense research. In a previous study, pooled analysis including 17 studies (16 case-control studies and 1 cohort study, mainly Caucasian) in the International Lung Cancer Consortium reported that pre-existing TB conferred an elevated overall risk of pulmonary cancer, with a relative risk (RR) of 1.48 and 95% confidence interval (CI) of 1.17 to 1.87.^[11]^ In another meta-analysis comprising 37 case-control studies and 4 cohort studies, focus was on the Chinese population and indicated significant lung cancer risk with previous TB exposure (RR = 1.74, 95% CI: 1.48–2.03).^[12]^ Furthermore, these results were verified in a cohort study performed in Taiwan using a large-scale population. In this study, a total of 716,872 cancer-free subjects were followed for 7 to 9 years, and it was found that the risk of developing lung cancer was 10.9-fold higher in those carrying a prior TB infection compared to their non-TB exposed counterparts, with a RR of 3.32 (95% CI: 2.70–4.09).^[13]^ These clinical or evidence-based studies strongly corroborate TB as a risk factor for lung cancer. In addition, excessive and persistent local inflammation in conjunction with lung fibrosis plays a critical part in pulmonary neoplasia.^[10]^ The prior TB episode of our patient increased her susceptibility to lung adenocarcinoma. Moreover, genetic alterations of β-catenin and inactivating mutations on adenomatous polyposis coli (APC) result in an aberrant nuclear/cytoplasmic expression of β-catenin in the morular component of the neoplasm due to decreased β-catenin degradation. Furthermore, driver genes, including cyclin D1 and c-myc will be activated and initiate tumor formation.^[14]^ Immunohistochemical staining for β-catenin was positive in the present case, thereby supporting the possible causal link between β-catenin expression and tumor formation.

Apart from differentiating lung carcinoid, pulmonary blastoma, and metastatic endometrioid carcinoma, WDFA needs to be distinguished from TB, which is critical in this case. From an imageological point of view, this type of tumor can be located in the bilateral lungs and commonly occurs in the left upper lobe, the right middle lobe, and the upper lobe, which is reminiscent of TB which is mainly located in the upper lobes of both lungs.^[15]^ If the patient's age at the time of onset of similar symptoms of TB is young, WDFA is often misdiagnosed when a chest radiograph is performed alone without a CT scan. According to the data presented in previous studies, the imaging of WDFA is non-specific. Most tumors are located under the pleura of the peripheral lung, and endobronchial involvement has also been reported.^[9,16]^ In CT images, WDFA is more likely to present typical features of lung cancer, such as burred signs, lobular signs, and strip signs, particularly lobular signs, while calcification appears more commonly in TB.^[17]^ Functionally, lesions with moderately high radiodensities on enhanced CT tend to be malignant, and inactive tuberculomas that display lower levels of enhancement are easily distinguished. Nevertheless, it lacks sufficient specificity to differentiate active inflammation.^[18]^

Most patients with WDFA are diagnosed during stage I, thus regional lymph nodes and distant metastases are less frequent.^[6]^ To date, complete surgical removal is the standard treatment for WDFA.^[9,19]^ Whether adjuvant therapy with chemoradiotherapy exerts a favorable effect warrants further investigation.^[19]^ Compared with stage-matched conventional adenocarcinoma of the lung, the prognosis of WDFA patients is usually better, with a 10-year survival of 75% or higher.^[20]^

## 4 Conclusion

To the best of our knowledge, this is the first case of WDFA that co-exists with TB. Although in previous studies concerning the uncommon tumor, it has been proposed that smoking increases the risk of WDFA; WDFA also occurs in never-smokers, and TB may be an underlying contributing factor. Thus, WDFA needs to be distinguished from not only lung carcinoid, pulmonary blastoma, metastatic endometrioid carcinoma, but also from TB. Although the condition is rare, WDFA should be considered in the differential diagnosis of confusing lung mass, especially in young females.

## Author contributions

**Data curation:** Haoyue Hu, Xiaoxiao Xie.

**Investigation:** Kuncheng Liu.

**Supervision:** Feng Luo.

**Writing - Original Draft:** Fengzhu Guo.

**Writing - Review & Editing:** Jiantao Wang.

Fengzhu Guo orcid: 0000-0002-0478-9315.
